# Supplementary material for: Complete Utilization of the Major Carbon Sources Present in Sugar Beet Pulp Hydrolysates by the Oleaginous Red Yeasts Rhodotorula toruloides and R. mucilaginosa
Source: J Fungi (Basel). 2021 Mar 17;7(3):215. doi: 10.3390/jof7030215 (PMC8002571; doi:10.3390/jof7030215)
Supplement: Supplementary file 1 [file jof-07-00215-s001.pdf]

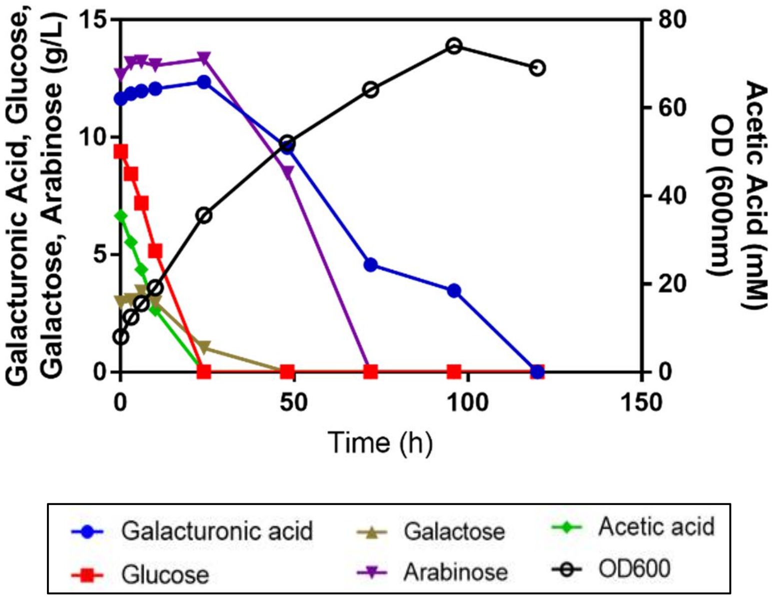

Figure S1- Growth and sugars utilization profiles by *R. mucilaginosa IST 390* cultivation in SBP hydrolysate H11 supplemented with commercial YNB containing 5 g/L of ammonium sulphate and amino acids (10 mg/L of L-histidine, 20 mg/L of DL-methionine and 20 mg/L DL-tryptophan). Medium was inoculated with an initial OD_{600nm} of 8 and was carried out at 30ºC, pH 5, with orbital agitation (250rpm).

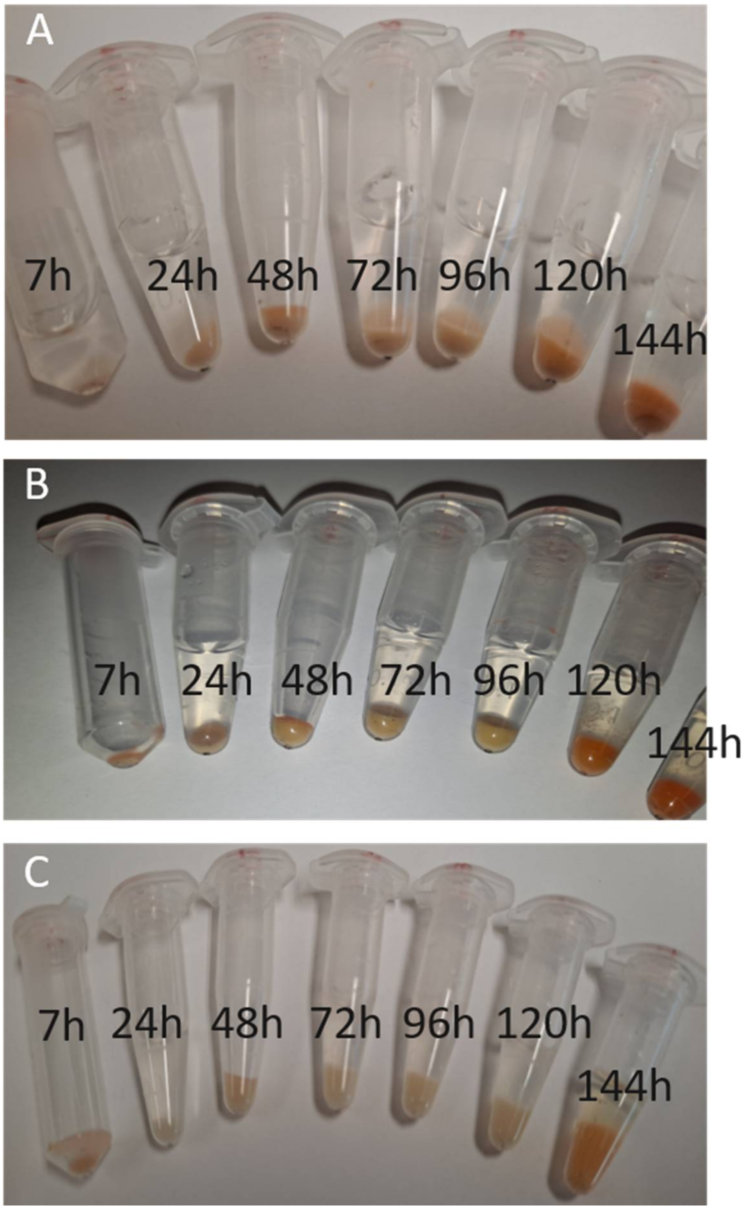

Figure S2- Cell pellets of *Rhodotorula mucilaginosa* IST 390 (A), *R. toruloides* PYCC 5615 (B) and *R. toruloides* IFO 0880 (C) harvested during yeast bioconversion of sugar beet pulp hydrolysate H13. Samples were harvested from the cultures characterized in Figure 6.
